# Differential expression and role of hyperglycemia induced oxidative stress in epigenetic regulation of β1, β2 and β3-adrenergic receptors in retinal endothelial cells

**DOI:** 10.1186/1755-8794-7-29

**Published:** 2014-05-30

**Authors:** Sher Zaman Safi, Rajes Qvist, Gracie Ong Siok Yan, Ikram Shah Bin Ismail

**Affiliations:** 1 Department of Medicine, Faculty of Medicine, University of Malaya, 50603 Kuala Lumpur, Malaysia; 2 Department of Anesthesiology, Faculty of Medicine, University of Malaya, 50603 Kuala Lumpur, Malaysia

**Keywords:** Expression, Methylation, ROS, Adrenergic receptors, Retinal endothelial cells

## Abstract

**Background:**

Aberrant epigenetic profiles are concomitant with a spectrum of developmental defects and diseases. Role of methylation is an increasingly accepted factor in the pathophysiology of diabetes and its associated complications. This study aims to examine the correlation between oxidative stress and methylation of β1, β2 and β3-adrenergic receptors and to analyze the differential variability in the expression of these genes under hyperglycemic conditions.

**Methods:**

Human retinal endothelial cells were cultured in CSC complete medium in normal (5 mM) or high (25 mM) glucose to mimic a diabetic condition. Reverse transcription PCR and Western Blotting were performed to examine the expression of β1, β2 and β3-adrenergic receptors. For detections, immunocytochemistry was used. Bisulfite sequencing method was used for promoter methylation analysis. Apoptosis was determined by the terminal deoxynucleotidyl transferase dUTP nick-end labeling (TUNEL) assay. Dichlorodihydrofluorescein diacetate (DCFH-DA) assay was used to measure reactive oxygen species (ROS) production in the cells.

**Results:**

β1 and β3-adrenergic receptors were expressed in retinal endothelial cells while β2-adrenergic receptor was not detectable both at protein and mRNA levels. Hyperglycemia had no significant effect on β1 and β2-adrenergic receptors methylation and expression however β3-adrenergic receptors showed a significantly higher expression (*p <* 0.05) and methylation (*p <* 0.01) in high and low glucose concentration respectively. Apoptosis and oxidative stress were inversely correlated with β3-adrenergic receptors methylation with no significant effect on β1 and β2-adrenergic receptors. β2-adrenergic receptor was hypermethylated with halted expression.

**Conclusion:**

Our study demonstrates that β1 and β3-adrenergic receptors expressed in human retinal endothelial cells. Oxidative stress and apoptosis are inversely proportional to the extent of promoter methylation, suggesting that methylation loss might be due to oxidative stress-induced DNA damage.

## Background

Diabetes is a growing epidemic, caused by excess glucose levels and body’s inability to produce or regulate insulin [1]. It is predicted that the number of people with diabetes will increase from 171 million in 2000 to 366 million by 2030 [2,3]. Diabetes is linked to several vascular pathologies, including severe blindness, atherosclerosis, stroke and renal failure. The growing number of people with diabetes suggests that diabetic retinopathy (DR) and diabetic macular edema (DME) will continue to be sight threatening diseases. Distinct morphological abnormalities in the retinal microvasculature either remain stable or progress to diabetic macular edema or proliferative diabetic retinopathy, the leading causes of severe visual impairment in working-age adults in industrialized countries [4,5].

In vitro and in vivo studies have revealed that hyperglycemic environment induces a number of cellular changes which affect the function and viability of cells [6,7]. Changes in the diabetic retina are due to a variety of factors including high glucose, oxidative stress and high levels of inflammatory markers [8,9]. Reactive oxygen species (ROS), especially mitochondrial ROS, play a significant role in modulating the cellular redox status [10]. Excessive production of ROS and the impairment of the oxidant/antioxidant balance may in part underlie the pathogenesis of diabetes and its associated complications [11]. Actions of such key pathological mediators of diabetes can lead to dysregulated epigenetic mechanisms that affect chromatin structure and gene expression profiles [12]. DNA methylation is one of the mechanisms for the epigenetic control of gene expression and aberrant DNA methylation patterns of CpG islands can influence normal transcriptional regulation in various diseases [13].

Beta adrenergic receptors are G protein coupled receptors (GPCRs), initially characterized by Ahlquist in 1948. [14]. Activation of these receptors takes place at the transmembrane region, which allows ligand binding and elicit a range of cellular actions such as phosphorylation and activation of various signaling pathways [15]. Upon binding of specific ligands, β1 and β2-adrenergic receptors activate the stimulatory G protein resulting in the dissociation of G protein subunits αβ from γ. The αβ subunits are linked to the stimulation of intracellular adenylyl cyclase, followed by the conversion of adenosine triphosphate into cyclic adenosine monophosphate (cAMP) and consequently leads to activation of protein kinase A and phosphorylation of several other substrates [16,17]. However contrary to β1 and β2-adrenergic receptors, β3-adrenergic receptor couple through the inhibitory G protein (Gi), causing a reduced generation of cAMP [18]. Work in animal models suggests that adrenergic receptor agents may promote corneal wound healing and it is known that cornea has an abundance of β-adrenergic receptors in both the epithelium and endothelium of the cornea [19]. Previous studies demonstrate that β-adrenergic receptors agonists have positive effects on the retina by reducing the inflammatory markers [20-22]. Studies on rats have demonstrated that highly selective agonists for β-ARs partially reversed obesity [23] and insulin resistance [24]. Similarly β-adrenergic receptor agonists like isoproterenol inhibits the formation of degenerative capillaries, prevents apoptosis of cells and reduces tumor necrosis factor (TNF) while anatagonists increase retinal dysfunction [25]. The role of Beta-adrenergic receptor agonists and antagonists in the treatments of glaucoma, diabetic retinopathy, and their potential mechanisms of actions, are still under investigation.

Type 2 diabetes is a multifactorial disease caused by a number of genetic, epigenetic and environmental factors [26]. In mammalian cells, DNA methylation takes place at cytosine in CpG dinucleotides and has been associated with transcriptional silencing [27]. Recent studies have shown that epigenetic modifications such as DNA methylation and histone modifications may affect the pathogenesis of type 2 diabetes [28,29]. Differential gene expressions show dynamic alterations in gene transcription and mRNA stability that can be influenced by the epigenetic modification of the genome in response to chronic hyperglycemic stress [30,31]. A number of studies have evaluated the epigenetic mechanisms in various developmental defects and diseases. Oxidative stress and alterations in DNA methylation have also been observed in diabetes, but no clear correlation between these events has been demonstrated until now. Very little research has focused on the epigenetics of adrenergic receptors in the eye and its relation to diabetic retinopathy. In the present study, we sought to determine which subtypes of Beta-adrenergic receptors that were expressed in retinal endothelial cells, and assessed the hyperglycemia induced differential expression. In addition we analyzed the role of oxidative stress modulating CpG methylation in the promoters of β1, β2 and β3-adrenergic receptors.

## Methods

### Cell culture

Human retinal endothelial cells (Cell Systems, ACBRI 181) were cultured in CSC complete medium (Cell Systems, 4Z0-500) supplemented with 10% serum, 1% Penicillin Streptomycin and animal derived growth factors. Cells were grown in attachment factor-coated dishes containing normal (5 mM, 0.9008 g/L) and high (25 mM, 4.5 g/L) glucose. When the cell reached 80% confluence, they were passaged with the use of a passage reagent group, supplied with the complete medium kit (Cell systems). The cells between 3-6 passages were used in all experiments and grown in 5% CO2 at 37°C. Media was changed every 2 to 3 days.

### Immunocytochemistry

The beta-adrenergic receptors were identified as described earlier [32], with minor changes. Retinal endothelial cells were seeded (50,000/well) onto 6-well plates with sterile coverslips in medium containing 5 mM (normal glucose) and 25 mM (high glucose). They were allowed to attach and proliferate in the respective medium for 2 days. The cells were fixed with cold methanol for 10 minutes at -20°C, rinsed twice with PBS, blocked at room temperature with 0.5% Tween (v/v) and 2% BSA (w/v) in PBS (blocking solution) for 1 hour and rinsed again twice with PBS. The wells were incubated overnight with primary antibodies against β1 adrenergic receptor (Santa Cruz, sc-568), β2-adrenergic receptor (Santa Cruz, sc-569) and β3-adrenergic receptor (Santa Cruz, sc-1472) in blocking solution at a dilution of 1:100. Anti-β-actin (sc-7210) antibodies were used as positive control. The following day, the cells were washed twice with PBS and incubated for 1 hour in dark room with secondary antibodies conjugated with fluorescein isothiocyanate (FITC) in blocking solution at a dilution of 1:200. After rinsing twice with PBS, the coverslips were mounted with antifading medium (Life Technologies, ProLong Gold) and analyzed under fluorescence microscope.

### RT PCR

Total RNA from Human retinal endothelial cells (HREC) was isolated using RNeasy commercial kit (Qiagen, cat no 74104) according to the manufacturer’s protocol and quantified spectrophotometrically. First-strand cDNA was synthesized with 1 μg of each DNA free total RNA, using iScript reverse transcription supermix (Biorad USA, cat no 170-8841) containing Moloney murine leukemia virus reverse transcriptase, RNase inhibitor, dNTPs, oligo-(dT), random primers, buffer, MgCl2 and stabilizers. No-RT control with the same amount of RNA but without RT was included to check genomic DNA carryover in RNA. Reaction conditions for cDNA synthesis were 25°C for 5 minutes, 42°C for 50 minutes and 85°C for 5 minutes. Equal amounts of cDNA were subsequently used for amplification in a 50 μl PCR reaction using EmeraldAmp PCR Master Mix (Takara, Japan), containing Taq polymerase, dNTPs, buffer and primers targeting β1-adrenergic receptor, β2-adrenergic receptor and β3-adrenergic receptor. PCR was performed with an initial denaturation step at 94°C for 3 minutes, followed by denaturation at 94°C for 30 seconds, annealing (60°C for β1-AR; 58°C for β2-AR; 61°C for β3-AR each for 30 seconds) and extension at 72°C for 1 minute. GAPDH was used as quantitative control in each PCR reaction. The PCR products were separated on 1.5% agarose gels and visualized using ethidium bromide staining under UV transillumination. Primers sequences of β1-AR, β2-AR and β3-AR are given in the Table 1.

**Table 1:** RT-PCR and methylation primers

**Gene names**	**RT-PCR primers**	**Methylation primers**
**β1-AR**	F: 5′-GGCAGCTGCTATTTCTGTCC-3′	F: 5′-GGATTGGTTGTAGGAGTTTGA-3′
	R: 5′-TCTGGACCAGTTCTGCCTCT-3′	R: 5′-TAAAAAACACCCCAAAAACCC-3′
**β2-AR**	F: 5′-ACAAACTATCCAGCAGATGAAAGG-3′	F: 5′-GGGTTAGTTTTAGGAGAAGGAGG-3′
	R: 5′-AGCGGGGGTATGCAAGTATG-3′	R: 5′-ATACCTACCAATTCCAACCC-3′
**β3-AR**	F: 5′-CCCACTTTCCCTCCGTTTGT-3′	F: 5′-GATTTGGTGATGGGATTTTTG-3′
	R: 5′-GAGTTTCAGGAAGGGTGGGG-3′	R: 5′-CCACCACTAACTCATAATAAAC-3′

### Western Blot analysis

Human retinal endothelial cells were lysed, centrifuged and proteins were extracted according to the manufacturer’s protocol (Sigma). Bradford assay (Bio-Rad Laboratories) was used to quantify the protein concentrations in the supernatants. 30 μg proteins of the lysate from high glucose and low glucose were loaded in each well and separated on 10% SDS-PAGE (Precast gels, Bio-Rad, cat no 456-1093). Gels were electrophoretically transferred to nitrocellulose membranes and blocked for 60 min with Tris-buffered saline (TBS) containing 5% nonfat milk (Bio-Rad, USA, cat no 170-6404) and 0.1% Tween-20. Blots were incubated overnight at 4°C with primary antibodies (1:3000) against β1-adrenergic receptor (Santa Cruz, sc-568), β2-adrenergic receptor (Santa Cruz, sc-569) and β3-adrenergic receptor (Santa Cruz, sc-1472). Anti-β-actin (sc-7210) antibodies were used to ensure the quality of protein separation and loading contents. After extensive washes, the membranes were incubated with HRP-conjugated IgG secondary antibodies (Santa Cruz, sc-2004) and visualized with enhanced chemiluminescence (Amersham Life Sciences, UK) using gel imaging system (Biospectrum 410, UVP).

### Terminal deoxynucleotidyl transferase dUTP nick end labeling (TUNEL) assay

DNA damage was investigated and quantified with a colorimetric apoptosis detection kit (Titer TACS; R&D System) according to the manufacturer’s protocol. Kit contained TUNEL staining in a 96-well format. Retinal endothelial cells were cultured in medium containing 5 mM (normal) and 25 mM (high) glucose. Cells were then transferred into a 96-well plate (1 × 10^5^ cells/well) and fixed with 3.7% buffered formaldehyde for 5 minutes followed by washing with PBS. Washing was followed by permeabilization of cells with 100% methanol for 20 minutes (at room temperature) and again washing with PBS. Following the manufacturer’s instructions, cells were then subjected to labeling procedure and the reaction was stopped with 0.2 N HCl after 30 minutes. Nuclease treated control was used to confirm the permeabilization and labeling reaction. The absorbance was measured at 450 nm with microplate reader.

### Dichloro-dihydro-fluorescein diacetate (DCFH-DA) assay

The intracellular ROS (reactive oxygen species) was measured by using 2′7′-dichloro-dihydro-fluorescin diacetate (DCFH-DA) reagent. The DCFH-DA enters through the cell membrane and enzymatically hydrolyzed by intracellular esterases to non-fluorescent dichlorofluorescin-diacetate (DCFHDA), which is then oxidized to highly fluorescent dichlorofluorescin (DCF) in the presence of intracellular reactive oxygen species. Retinal endothelial cells were seeded in 96-well black plates and allowed to grow for 24 hours. Cells were incubated for an additional 24 hours in media containing 5 mM (normal) and 25 mM (high) glucose. Cells were then treated with 5 μM DCFH-DA and the readings were taken at 485 nm excitation and 530 nm emission in a fluorescence plate reader. To calculate the amount of intracellular ROS produced, the mean control was subtracted from the mean experimental group.

### 8-OHdG ELISA assay

8-Hydroxy-2′-deoxyguanosine (8-OHdG), one of the DNA lesions has been studied extensively as a marker of oxidative stress-mediated DNA damage, was estimated in retinal endothelial cells treated with high glucose, normal glucose and H2O2 as a control, using Oxiselect oxidative DNA damage ELISA kit (Cell Biolabs). Cells were grown and treated with high glucose (25 mM), normal glucose (5 mM) and H2O2 (50 mM) as a control. The whole experiment was performed as previously reported with minor modifications [33].

### Selection of upstream and downstream sequences within CpG Islands

**Figure 1:**
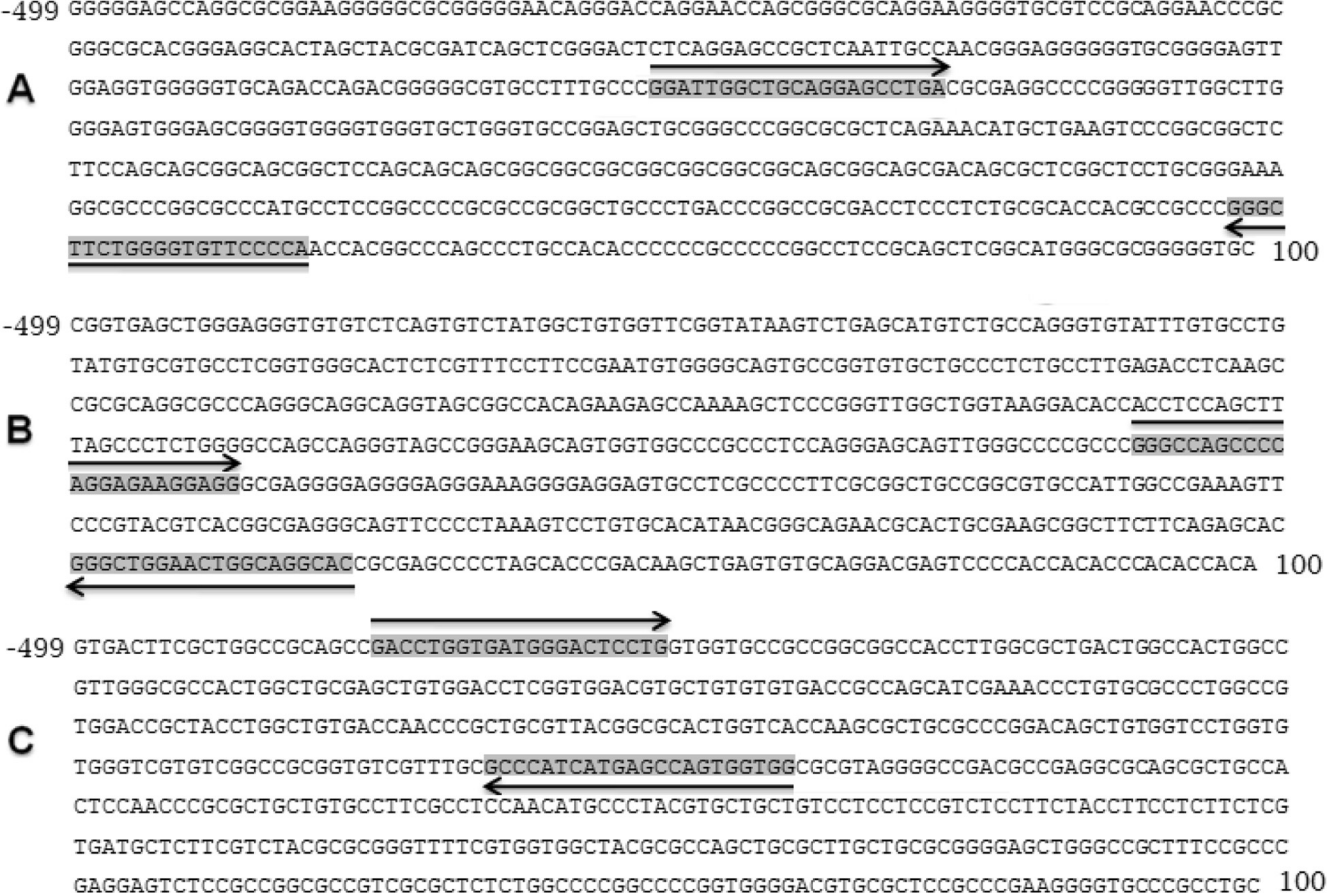
**Promoter DNA sequences of β1, β2 and β3-adrenergic receptors from -499 to 100 bp.** Relative to CpG contents and transcription start sites, sequences were retrieved from Eukaryotic Promoter Database (EPD). Arrows indicate forward and reverse primers that amplify regions containing 40 CpGs in β1-adrenergic receptor **(A)**, 16 CpGs in β2-adrenergic receptor **(B)** and 27 CpGs in β3-adrenergic receptor **(C)**.

DNA sequences of CpG islands in β1-adrenergic receptor (2161 bp), β2-adrenergic receptor (807 bp) and β3-adrenergic receptor (1522 bp) were retrieved from NCBI and UCSC genome browser. Promoter region of each gene from upstream -499 bp to downstream 100 bp, relative to CpG contents and transcription start sites, were identified using Eukaryotic Promoter Database (EPD). Subsequently, primers were designed within -499 to 100 bp region of each promoter with products containing 40 CpGs in β1-adrenergic receptor , 16 CpGs in β2-adrenergic receptor and 27 CpGs in β3-adrenergic receptor (Figure 1A, B and C).

### DNA extraction, bisulfite treatment and PCR amplification

DNA was extracted from retinal endothelial cells grown in high and normal glucose using genomic DNA mini kit (Invitrogen USA, Cat no K1820-02) according to the manufacturer’s protocol. 500 ng of DNA was bisulfite-converted with Zymo EZ DNA methylation kit (cat no D5005, USA). PCR was used to amplify the bisulfite modified DNA using hot start taq DNA polymerase and bisulfite primers targeting the promoters of β1, β2 and β3-adrenergic receptors. PCR reaction conditions were set as 5 minutes at 95°C; 40 seconds at 95°C, 1 minute at 60°C and 1 minute at 72°C; and a 7 minutes final extension at 72°C. PCR products were analyzed on 2% agarose gel and purified using mini elute gel extraction kit (Qiagen, Cat no 28604) according to the manufacturer’s instructions.

### Cloning and transformation

The purified products were ligated into pCR2.1 vector using TOPO TA Cloning Kit (Invetrogin). 4 μl of PCR product and 1 μl of TOPO vector, with a final volume of 6 μl were gently mixed and incubated at room temperature for 5 minutes. Cloning reaction was added to chemically competent DH5α, mixed and incubated on ice for 10 minutes. Then subjected the cells to heat shock the cells for 30 seconds at 42°C and again transferred to ice. 250 μl S.O.C medium was added (provided with kit, invitrogen), spread each transformation onto pre-warmed LB plates (ImMedia Amp Blue, Invitrogen USA) and incubated overnight at 37°C. pUC19 DNA was used as control.

### Sequencing and Methylation analysis

**Figure 2:**
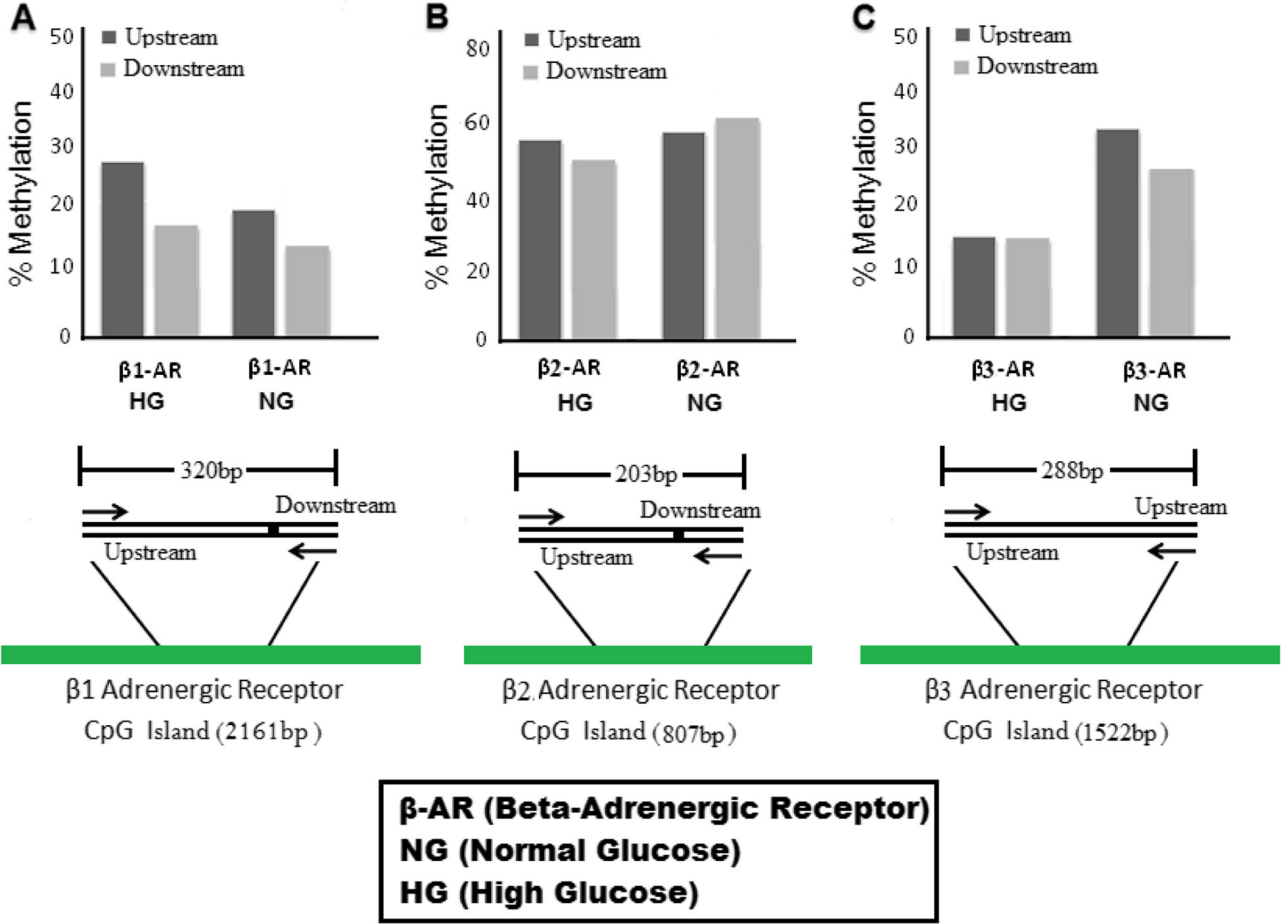
**Percent methylation of upstream and downstream sequences.** CpG islands and the sites which were amplified, bisulfite converted and sequenced for β1-adrenergic receptor **(A)**, β2-adrenergic receptor **(B)** and β3-adrenergic receptor **(C)**.

After blue-white screening, colonies (10 colonies for each β1, β2 and β3-adrenergic receptors) containing the insert were isolated and cultured overnight in ImMedia Amp Liquid (Invitrogen, USA). Favorgen plasmid extraction kit (cat no FAPDE100) was used to purify the plasmids from each colony, which were then sent for sequencing. After sequencing, UCSC genome browser, NCBI, Ensembl and QUMA (Quantification tool for methylation analysis) were used to align, visualize and quantify the bisulfite sequence data for CpG methylation of β1, β2 and β3-adrenergic receptors. The regions examined in all genes were located within CpG islands as shown in the Figure 2A, B and C.

### Statistical analysis

All experiments were performed at least 3 times. The results were reported as mean SD. Student t test was used to determine statistical significance. A value of 0.05 was considered as significant. SPSS and graphpad prism softwares were used for analysis.

## Results

### Adrenergic receptors β1 and β3 expression in retinal endothelial cells

**Figure 3:**
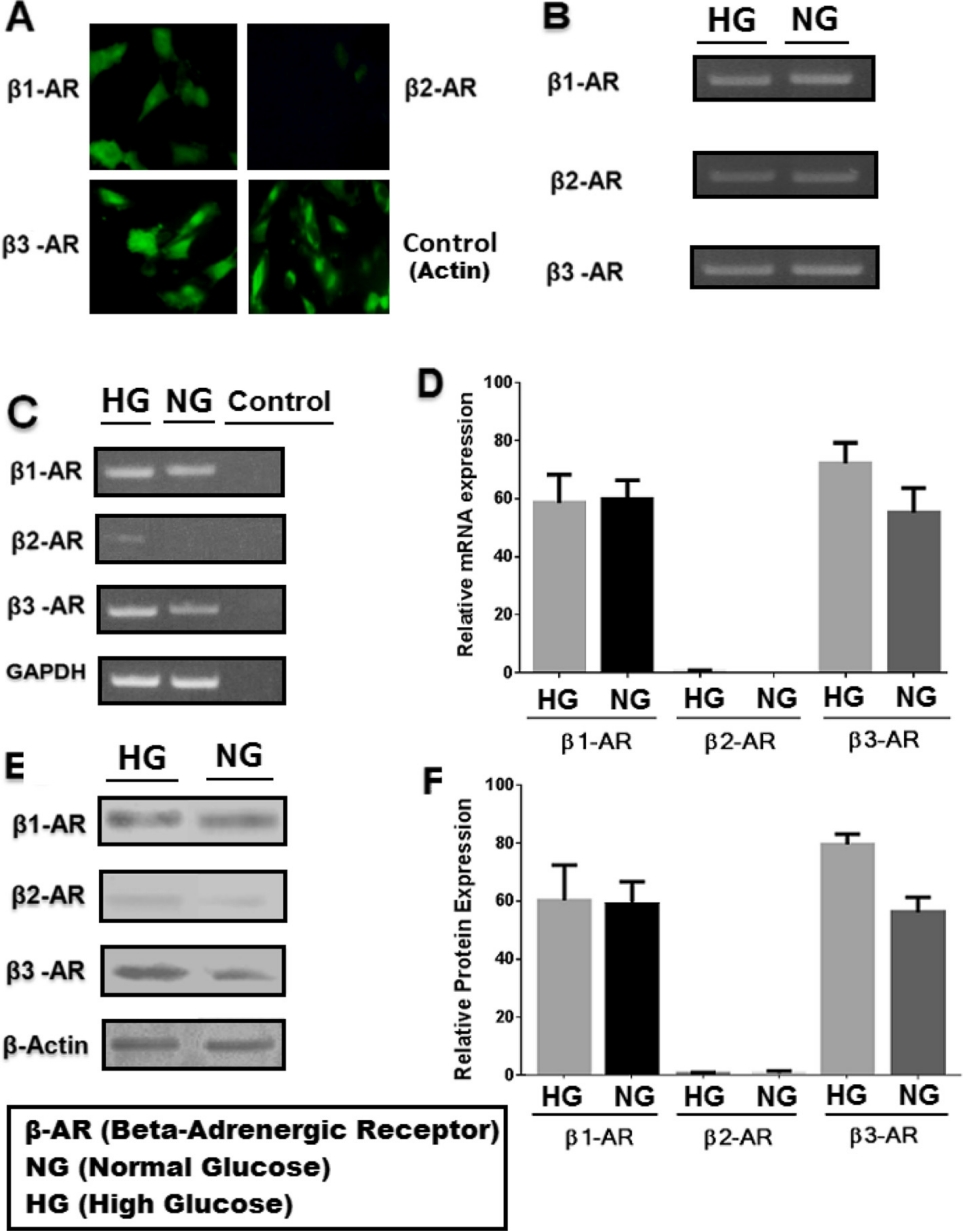
**Amplification of CT converted DNA and expression analysis. (A)** Immunocytochemistry of β-adrenergic receptors, showing strong expression of β1and β3-adrenergic receptors. **(B)** Amplification of CT converted DNA using bisulfite converted primers for β1, β2 and β3-adrenergic receptors. **(C)** Expression levels of β1, β2 and β3-adrenergic receptors genes by reverse transcription, in high and normal glucose conditions. GAPDH was used as a loading control while cDNA without Reverse Transcriptase was used as negative control. **(D)** Graphical presentation of β1, β2 and β3-adrenergic receptors expression of mRNA **(E)** Expression of β1, β2 and β3-adrenergic receptors at protein level by Western Blotting in high and normal glucose. **(F)** Graphical presentation of relative expression of β1, β2 and β3-adrenergic receptors genes in retinal endothelial genes.

Adrenergic receptors β1 and β3 were expressed in retinal endothelial cells both under high and normal glucose conditions. First immunocytochemistry was carried out (Figure 3A) and then reverse transcription was performed to confirm the presence of β1, β2 and β3-adrenergic receptors which revealed no expression of β2-adrenergic receptor but relatively high expression of β1 and β3-adrenergic receptors (Figure 3C and D). To reconfirm the expression of target genes, mRNA measurement by reverse transcription was followed by Western Blotting. In Western Blotting analysis, β1 and β3-adrenergic receptors were expressed in the same manner as previously detected by RT-PCR analysis (Figure 3E and F).

### β3-Adrenergic receptor is differentially expressed in retinal endothelial cells under hyperglycemic conditions

Gene expression of β1, β2 and β3-adrenergic receptors were compared within the same endothelial cells, grown in high (25 mM) and normal (5 mM) glucose levels. Expression of β3-adrenergic receptor in high glucose was significantly (*p <* 0.05) different than the expression of the same gene in normal glucose at both protein and mRNA levels (Figure 3C, D, E and F).

### Adrenergic receptor β1 is not differentially expressed in retinal endothelial cells while β2-adrenergic receptor is not detectable both under normal and high glucose levels

According to the results obtained from protein and mRNA analysis, the expression of β1-adrenergic receptor was not significantly different both in high and normal glucose conditions (p = 0.8544). β2-adrenergic receptor was not detectable at mRNA level as well as protein level, however very small and defused bands were seen when retinal endothelial cells were grown under hyperglycemic condition (Figure 3C and E).

### Promoter methylation and its association with the expression of β1, β2 and β3-adrenergic receptors in retinal endothelial cells

**Figure 4:**
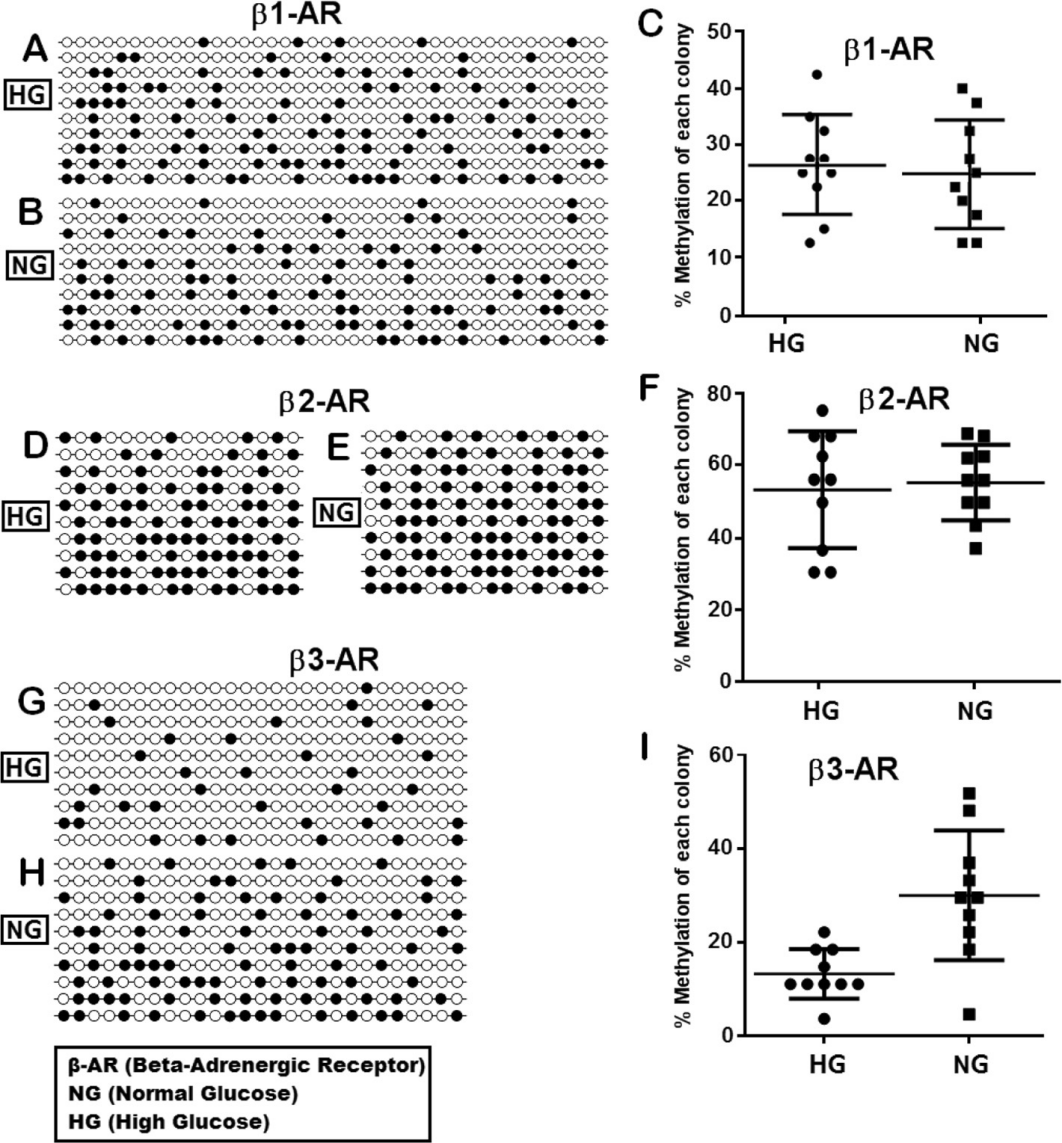
**Methylation analyses.** Methylation (black dots) of β1-adrenergic receptor (**A** and **B** in high and normal glucose respectively), β2-adrenergic receptor (**D** and **E** in high and normal glucose respectively) and β3-adrenergic receptor (**G** and **H** in high and normal glucose respectively), using QUMA (Quantification tool for methylation analysis). Empty **C**, **F** and **I** show methylation of each colony.

**Figure 5:**
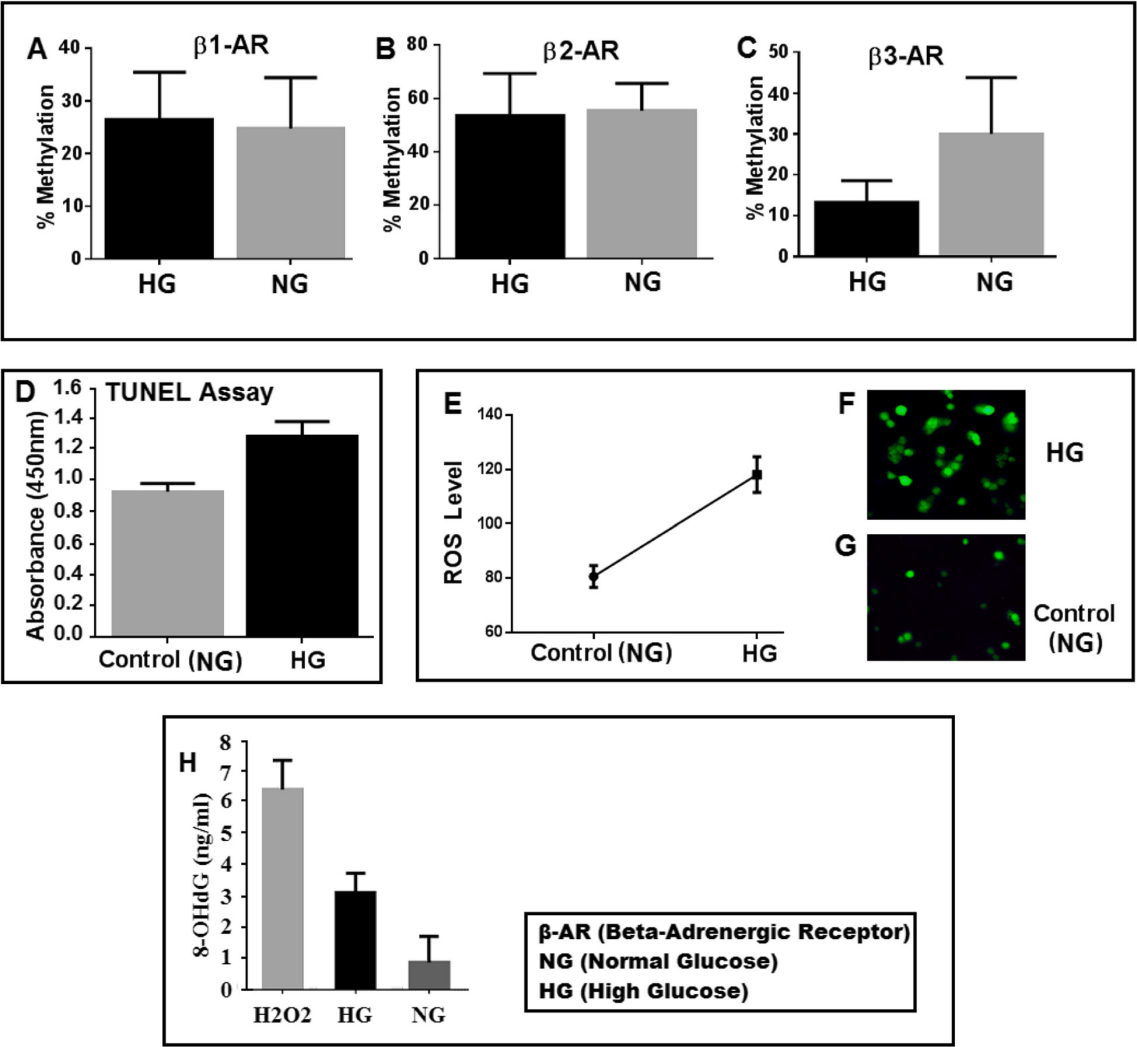
**Percent methylation of β1-AR (A), β2-AR (B) and β3-AR (C).** β3-AR shows a significantly higher methylation in normal glucose conditions (*p <* 0.01). **(D)** shows terminal deoxynucleotidyl transferase dUTP nick-end labeling (TUNEL) assay that demonstrates a significantly high (*p <* 0.005) apoptosis of retinal endothelial cells grown in hyperglycemia. **(E, F and G)** show Dichlorodihydrofluorescein diacetate (DCFH-DA) assay showing a high production (*p <* 0.001) of reactive oxygen species (ROS) in retinal endothelial cells that were grown in high glucose (25 mM) condition **(F)**, while low production in cells grown in normal glucose (5 mM) condition **(G)**. 8-OHdG level in high and normal glucose **(H)** where H2O2 was used as control.

**Figure 6:**
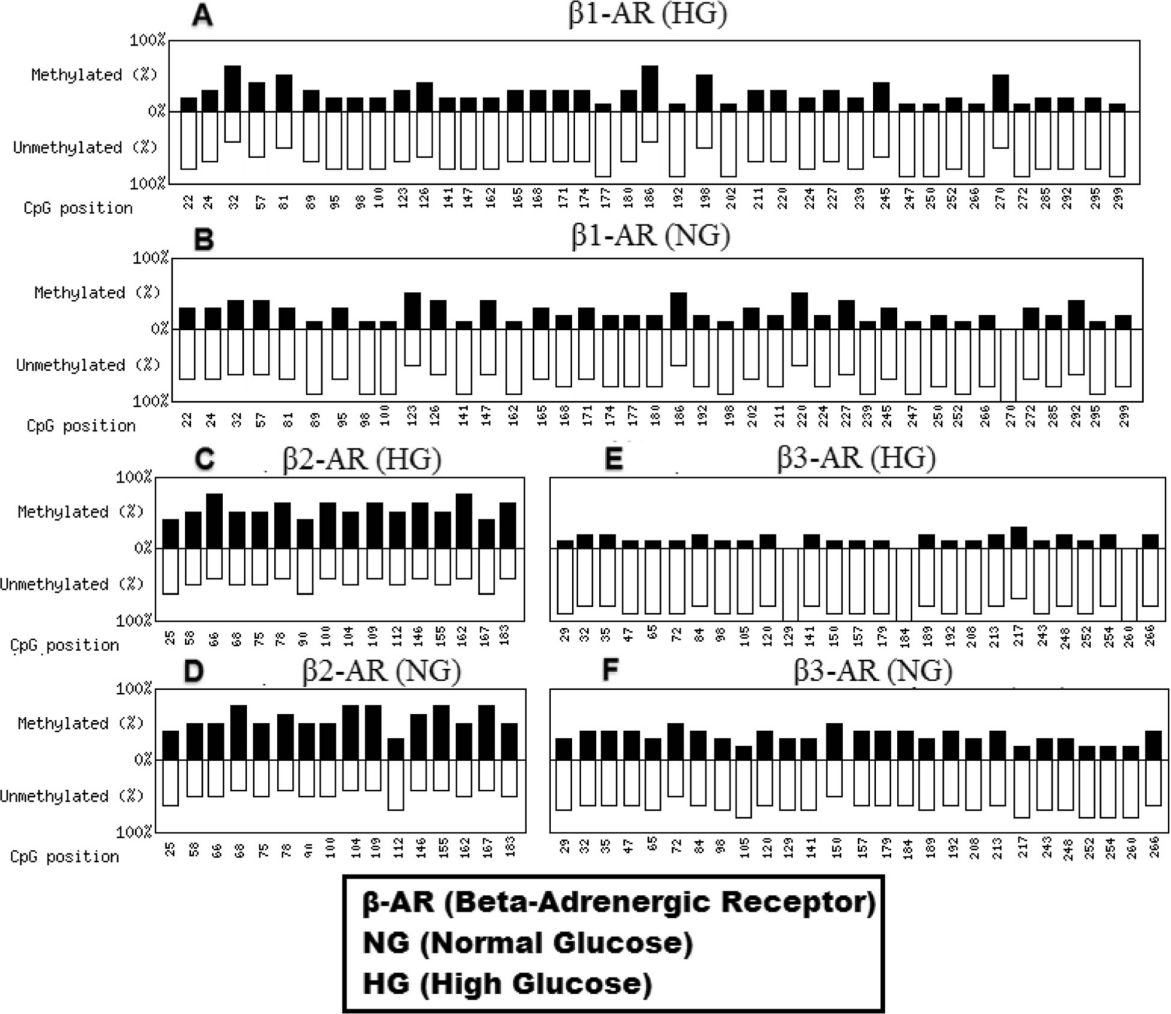
**Graphical presentation of CpGs in 10 samples of each gene.** Black bars show methylated while white bar show unmethylated CpGs at a certain position. In β1-adrenergic receptor there are 40 CpGs as shown by **A** (high glucose) and **B** (lnormal glucose) while in β2-adrenergic receptor there are 16 CpGs (**C** and **D**, high glucose and normal glucose respectively). **E** and **F** show methylated and unmethylated CpGs in β3-adrenergic receptor in high and normal glucos respectively.

According to our data, β1-adrenergic receptor showed no significant difference in promoter methylation when cells were grown in either high or normal glucose levels. We evaluated 40 CpGs in the promoter of β1-adrenergic receptor which showed 26.5% methylation in high glucose and 24.5% methylation in normal glucose (Figure 4A, B and C). Our results indicate no significant association of promoter methylation and expression of β1-adrenergic receptor in human retinal endothelial cells cultured under hyperglycemic conditions (Figure 5A). β2-adrenergic receptor showed 53.7% and 55% methylation in high and normal glucose respectively. Although the difference in high and normal glucose did not achieve significance, hypermethylated promoter patterns show that β2-adrenergic receptor gene is silenced in retinal endothelial cells possibly due to hypermethylation of its promoter (Figure 4D, E and F). Promoter methylation of β3-adrenergic receptor was significantly different (*p <* 0.01) with 13.3% and 33.7% methylation in high and normal glucose respectively, having an inverse correlation with its expression (Figure 4G, H and I). Interestingly the expression of β3-adrenergic receptor was also significantly different both at mRNA (*p <* 0.05) and protein (*p <* 0.01) levels in high and normal glucose conditions. Total 27 CpGs were included in from the promoter of β3-adrenergic receptor.A graphical presentation of CpGs in 10 samples of each gene is shown in Figure 6 with black bars for methylated and white bars for unmethylated CpGs at a certain position. β1-adrenergic receptor shows maximum CpG methylation at position 32, 186, 198 and 270 in high glucose (Figure 6A) while in normal glucose the high occurrence of methylation was seen at position 123, 186 and 220(Figure 6B). In β2-adrenergic receptor methylation was high at position 66, 78, 100 and 162 (Figure 6C) and 68, 104, 109, 155 and 167 (Figure 6D) in high and normal glucose respectively. β3-adrenergic receptor revealed high methylation at position 217 in high glucose while at position 72 and 150 in normal glucose.

### Oxidative stress, apoptosis and promoter methylation of β1, β2 and β3-adrenergic receptors in retinal endothelial cells

Reactive oxygen species (ROS) production was significantly high (*p <* 0.001) in retinal cells grown in high glucose (25 mM) compared to normal (5 mM) glucose concentration as shown in Figure 5E, F and G. ROS production in high glucose was inversely proportional to methylation of β3-adrenergic receptor promoter in high glucose, suggesting that DNA damage by increased ROS production may induce loss of methylation under hyperglycemic condition. β2-adrenergic receptor showed the same pattern of methylation and ROS production in high glucose; however CpG methylation in high and normal glucose was not significantly different (Figures 4D and 5B). Apoptosis in high glucose was also significantly higher (*p <* 0.005) as compared to normal glucose and was positively correlated with the extent of ROS production. Diabetes mellitus is associated with oxidative stress, leading to protein, lipid and DNA modifications. Oxidative DNA damage is usually evaluated by measuring 8-OHdG (8-hydroxy-2-deoxy-guanosine). In our results 8-OHdG level was significantly (*p <* 0.05) higher in high glucose as compared to normal glucose. For β3-adrenergic receptor, DNA damage was inversely proportional to the level of CpG methylation in retinal endothelial cells (Figure 5C, F and H).

### Upstream and downstream promoter methylation of adrenergic receptors is not significantly different except β1-adrenergic receptor in high glucose and β3-adrenergic receptor in normal glucose

Over all in this study, no significant association was found in methylation between upstream and downstream TSS except β1-adrenergic receptor in high glucose (*p <* 0.005) and β3-adrenergic receptor in normal glucose (*p <* 0.005). Upstream and downstream promoter methylation of β1-adrenergic receptor were 27.2% and 16.6% in high glucose, while 18.1% and 13.3% in normal glucose respectively. β2-adrenergic receptor showed 54.6% and 50% methylation values in high glucose while 55.1.7% and 60.6% methylation in normal glucose at upstream and downstream CpGs respectively. At upstream and downstream promoter sequences, methylation level of β3-adrenergic receptor were 13.3% and 13.4% in high glucose, while 34.1% and 26.3% in normal glucose respectively.

## Discussion

Diabetes and diabetogenic agents such as high glucose and advanced glycation end-products have adverse effects in major target cells including endothelial cells and vascular smooth muscle cells [34-36]. In diabetic retinopathy hyperglycemia cause morphological and functional damage to the retina through a number of mechanisms including oxidative stress, proinflammatory cytokines and increased TNFa signaling [37-41]. According to the published reports, β-adrenergic receptors are likely to play a significant role in potentiating retinal endothelial cells against hyperglycemia and hyperglycemia-induced oxidative stress. The oxidative stress-induced damage is not limited to its direct effect on cellular components including DNA, protein, free fatty acids and lipids, but may extend to the ability of oxidative stress to alter gene expression. Thus we sought to investigate whether hyperglycemia induced oxidative stress and apoptosis can influence β-adrenergic receptors methylation and expression in retinal endothelial cells.

DNA methylation is an important genetic mechanism that plays an essential role in the regulation of gene expression in various diseases such as diabetes, cancer, muscular dystrophy and a range of birth defects, but a comprehensive understanding of the diversity of the mechanisms involved in methylation is not understood [42]. Despite limited data on the epigenetic regulation and its association with the disease progression, the underlying relationship between changes in promoter DNA methylation and differences in gene regulation are well established [43,44]. Previous studies have provided evidence which link gene methylation and transcription in a number of genes and tissue specific cell lines; however methylation studies on G protein-coupled receptors in general and on β-adrenergic receptors in particular are very limited. We know very little about the changes in epigenetic profiles of β-adrenergic receptors that might explain differences in gene expression. DNA methylation, especially CpG methylation at promoter regions, has been an important modification that prevents transcriptions factors (TF) recruitment and consequently suppresses the transcription of various genes. Promoter methylation is considered a key regulator of biological processes such as suppression of transposable elements, X-chromosome inactivation and genomic imprinting. CpG dependent transcription factor binding is a prominent feature and provides support to understand the role and mechanism of TFs in epigenetic regulation [45]. Various transcription factors including E2F, HIF-1, and c-myc bind to sequences containing CpGs in the promoters [46,47]. Methylation does not interfere only in transcription factor binding activity, but also modulates chromatin structure by modifying the interaction between core histones and DNA. Previous studies show that Sp1 binding to its cognate sequence in certain genes is affected by methylation [48]. Β-adrenergic receptors contain special sites such as TATA box, CAAT box, mRNA cap sites and GC rich sites where TF Sp1 binds [49]. In a recent study, Mu et al. found that β2-adrenergic receptor stimulation led to decreased transcription of the gene encoding WNK4. Stimulation of β2-adrenergic receptor caused cAMP-dependent inhibition of histone deacetylase-8 (HDAC8) activity, increased histone acetylation and consequently binding of the glucocorticoid receptor to a negative glucocorticoid responsive element in the promoter region [50]. For our results, we also hypothesize that hypermethylation of β2-adrenergic receptor might prevent the core transcription factors such as Sp1 to bind with its promoter and hence causing halted expression of β2-adrenergic receptor.

In our study, protein and mRNA expression of β1 and β3-adrenergic receptors were confirmed in retinal endothelial cells however β2-adrenergic receptor was hard to detect both at mRNA and protein levels. According to several studies including our previous unpublished data, hyperglycemia increases the oxidative stress in several cell lines. Kong et al. and Takahata et al. in two different studies found that upregulation of β3-adrenergic receptor is associated with increased oxidative stress [51] while functional expression of β2-adrenergic receptor is responsible for protection against oxidative stress through promotion of glutathione synthesis [52]. In 2003, Steinle et al. reported that β*3-*adrenergic receptor mediates migration and proliferation of retinal endothelial Cells. They Used BRL37344 agonist to stimulate the β_3_-adrenergic receptors on retinal endothelial cells and showed that these receptors could express in retinal endothelial cells. They reported that BRL37344-mediated migration could be alienated by prior inhibitors administration of Akt, PI3K, MEK and MMP-2/MMP-9 and hypothesized that PI3K may directly be linked to the β_3_-adrenergic receptor [53]. These studies led us to question whether our hyperglycemic endothelial cells with no agonist (BRL37344) and increased oxidative stress would differentially mediate the promoter methylation and expression of β3, β2 and β1-adrenergic receptors. In our study, hyperglycemic endothelial cells had significantly higher oxidative stress and consequently a higher expression of β3-adrenergic receptor. Methylation in β3-adrenergic receptor was significantly low in cells with significantly high apoptosis and oxidative stress as per our hypothesis.

There is an exquisite interrelationship between methylation and oxidative stress. Glutathione is depleted by the excess of oxidative stress; consequently impairing the one-carbon cycle and causes undermethylation. However on the other hand glutathione, cysteine, and metallothionein get depleted by undermethylation with oxidative stress overload. Increased production of ROS can directly or indirectly cause a wide range of DNA lesions including base modifications, strand breakage, deletions and chromosomal rearrangements. Such alterations have been shown to impede the ability of DNA to function as a substrate for the DNA methyl transferases (DNMTs), resulting in global hypomethylation [54-56]. In our data β3-adrenergic receptor is hypomethylated with increased expression. The reason behind hyperglycemia led increase in β3-adrenergic receptor is not clear however Walker and Steinle suggest that the level of norepinephrine are significantly reduced as early as 1 week after streptozotocin (STZ) treatment, this loss of norepinephrine may consequently lead to denervation supersensitivity and increased expression of β1-adrenergic receptor in Muller cells [33]. Hypermethylation and low expression of β2-adrenergic receptor is another interesting finding in our study. In our study we selected upstream and downstream regions, however high and normal glucose induced no variation and no association was seen in methylation between upstream and downstream sequences except β1-adrenergic receptor in high glucose and β3-adrenergic receptor in normal glucose.

## Conclusion

Our study demonstrates that β1 and β3-adrenergic receptors β-ARs expressed in human retinal endothelial cells HREC. Oxidative stress and apoptosis are inversely proportional to the extent of promoter methylation, suggesting that methylation loss might be due to oxidative stress-induced DNA damage. Collectively this study may help in understanding the pathophysiology of diabetic retinopathy and epigenetic regulation of β-adrenergic receptors.

## Competing interests

The authors declare that they have no competing interests.

## Authors’ contributions

SZS carried out the experiments and design of study. RQ helped in revising the manuscript and data analysis. The entire study was overseen by ISBI. All authors contributed to writing the manuscript and approved the final manuscript.

## Pre-publication history

The pre-publication history for this paper can be accessed here:

http://www.biomedcentral.com/1755-8794/7/29/prepub

## References

[B1] Newman JC He W Verdin E Mitochondrial protein acylation and intermediary metabolism: regulation by sirtuins and implications for metabolic disease J Biol Chem 2012 7 42463 42443 10.1074/jbc.R112.404863 PMC3522244 23086951

[B2] Wild S Roglic G Green A Sicree R King H Global prevalence of diabetes: estimates for the year 2000 and projections for 2030 Diabetes Care 2004 7 1047 1053 10.2337/diacare.27.5.1047 15111519

[B3] World Health Organization Diabetes Action Now, An initiative of the world health organization and the international diabetes federation 2004 Geneva: World Health Organization

[B4] Kempen JH O’Colmain BJ Leske MC Haffner SM Klein R Moss SE Taylor HR Hamman RF The prevalence of diabetic retinopathy among adults in the United States Arch Ophthalmol 2004 7 552 563 15078674 10.1001/archopht.122.4.552

[B5] Williams R Airey M Baxter H Forrester J Kennedy-Martin T Girach A Epidemiology of diabetic retinopathy and macular oedema: a systematic review Eye 2004 7 963 983 10.1038/sj.eye.6701476 15232600

[B6] Kowluru RA Diabetic Retinopathy: Mitochondrial Dysfunction and Retinal Capillary Cell Death Antioxid Redox Signal 2005 7 1581 10.1089/ars.2005.7.1581 16356121

[B7] Kowluru RA Kennedy A Therapeutic potential of anti-oxidants and diabetic retinopathy Expert Opin Investig Drugs 2001 7 1665 1676 10.1517/13543784.10.9.1665 11772276

[B8] El-Asrar AMA Dralands L Missotten L Al-Jadaan IA Geboes K Expression of Apoptosis Markers in the Retinas of Human Subjects with Diabetes Invest Ophthalmol Vis Sci 2004 7 2760 2766 10.1167/iovs.03-1392 15277502

[B9] Joussen AM Doehmen S Le ML TNF-alpha mediated apoptosis plays an important role in the development of early diabetic retinopathy and long-term histopathological alterations Mol Vis 2009 7 1418 1428 19641635 PMC2716944

[B10] Cheng X Siow RC Mann GE Impaired redox signaling and antioxidant gene expression in endothelial cells in diabetes: a role for mitochondria and the nuclear factor-E2-related factor 2-Kelch-like ECH-associated protein 1 defense pathway Antioxid Redox Signal 2011 7 469 487 10.1089/ars.2010.3283 20524845

[B11] Brownlee M Biochemistry and molecular cell biology of diabetic complications Nature 2001 7 813 820 10.1038/414813a 11742414

[B12] Reddy MA Natarajan R Epigenetic mechanisms in diabetic vascular complications Cardiovasc Res 2011 7 3 421 429 10.1093/cvr/cvr024 21266525 PMC3096305

[B13] Schinke C Mo Y Yu Y Amiri K Sosman J Greally J Verma A Aberrant DNA methylation in malignant melanoma Melanoma Res 2010 7 253 265 10.1097/CMR.0b013e328338a35a 20418788 PMC3026062

[B14] Bylund DB Eikenberg DC Hieble JP Langer SZ Lefkowitz RJ Minneman KP Molinoff PB Ruffolo RR Jr Trendelenburg U International Union of Pharmacology nomenclature of adrenoceptors Pharmacol Rev 1994 7 2 121 136 7938162

[B15] Chakraborti S Chakraborti T Shaw G [beta]-adrenergic mechanisms in cardiac diseases: A perspective Cell Signal 2000 7 499 513 10.1016/S0898-6568(00)00087-5 11027943

[B16] Stiles GL Caron MG Lefkowitz RJ Beta-adrenergic receptors: biochemical mechanisms of physiological regulation Physiol Rev 1984 7 661 743 6143332 10.1152/physrev.1984.64.2.661

[B17] Wenzel-Seifert K Liu HY Seifert R Similarities and differences in the coupling of human beta1- and beta2-adrenoceptors to Gs(alpha) splice variants Biochem Pharmacol 2002 7 9 20 10.1016/S0006-2952(02)00924-3 12106601

[B18] Gauthier C Tavernier G Charpentier F Langin D Le Marec H Functional beta3-adrenoceptor in the human heart J Clin Invest 1996 7 556 562 10.1172/JCI118823 8755668 PMC507461

[B19] Elena PP Denis P Kosina-Boix M Saraux H Lapalus P Beta adrenergic binding sites in the human eye: an autoradiographic study J Ocul Pharmacol 1990 7 2 143 149 10.1089/jop.1990.6.143 2168462

[B20] Steinle JJ Chin VC Williams KP Panjala SR Beta-adrenergic receptor stimulation modulates iNOS protein levels through p38 and ERK1/2 signaling in human retinal endothelial cells Exp Eye Res 2008 7 1 30 34 10.1016/j.exer.2008.04.008 18541234

[B21] Walker R Steinle J Role of beta-adrenergic receptors in inflammatory marker expression in muller cells Invest Ophthalmol Vis Sci 2007 7 11 5276 5281 10.1167/iovs.07-0129 17962483

[B22] Williams KP Steinle JJ Maintenance of beta-adrenergic receptor signaling can reduce Fas signaling in human retinal endothelial cells Exp Eye Res 2009 7 4 448 455 10.1016/j.exer.2009.04.015 19523948

[B23] Ghorbani M Himms-Hagen J Appearance of brown adipocytes in white adipose tissue during CL 316,243-induced reversal of obesity and diabetes in Zucker fa/fa rats Int J Obes 1997 7 465 475 10.1038/sj.ijo.0800432 9192230

[B24] Largis E Burns M Muenkel H Dolan J Claus T Antidiabetic and antiobesity effects of a highly selective ß3-adrenoceptor agonist (CL 316,243) Drug Dev Res 1994 7 32 69 76

[B25] Jiang Y Steinle JJ Systemic propranolol reduces b-wave amplitude in the ERG and increases IGF-1 receptor phosphorylation in rat retina Invest Ophthalmol Vis Sci 2010 7 5 2730 2735 10.1167/iovs.09-4779 20042659 PMC2868483

[B26] Ling C Groop L Epigenetics: a molecular link between environmental factors and type 2 diabetes Diabetes 2009 7 2718 2725 10.2337/db09-1003 19940235 PMC2780862

[B27] Lister R Pelizzola M Dowen RH Hawkins RD Hon G Tonti-Filippini J Nery JR Lee L Ye Z Ngo QM Edsall L Antosiewicz-Bourget J Stewart R Ruotti V Millar AH Thomson JA Ren B Ecker JR Human DNA methylomes at base resolution show widespread epigenomic differences Nature 2009 7 7271 315 322 10.1038/nature08514 19829295 PMC2857523

[B28] Ling C Del Guerra S Lupi R Rönn T Granhall C Luthman H Masiello P Marchetti P Groop L Del Prato S Epigenetic regulation of PPARGC1A in human type 2 diabetic islets and effect on insulin secretion Diabetologia 2008 7 4 615 622 10.1007/s00125-007-0916-5 18270681 PMC2270364

[B29] Rönn T Poulsen P Hansson O Holmkvist J Almgren P Nilsson P Tuomi T Isomaa B Groop L Vaag A Ling C Age influences DNA methylation and gene expression of COX7A1 in human skeletal muscle Diabetologia 2008 7 7 1159 1168 10.1007/s00125-008-1018-8 18488190

[B30] Ekstrom TJ Stenvinkel P The epigenetic conductor: a genomic orchestrator in chronic kidney disease complications? J Nephrol 2009 7 442 449 19662598

[B31] Ingrosso D Perna AF Epigenetics in hyperhomocysteinemic states. A special focus on uremia Biochim Biophys Acta 2009 7 892 899 10.1016/j.bbagen.2008.11.010 19245874

[B32] Castillo C Albasanz J Fernandez M Martin M Endogenous expression of adenosine receptors in rat C6 glioma cells Neurochem Res 2007 7 1056 1070 10.1007/s11064-006-9273-x 17401671

[B33] Walkernand RJ Steinle JJ Role of β-Adrenergic Receptors in Inflammatory Marker Expression in Muller Cells Invest Ophthalmol Vis Sci 2007 7 1 1 40 10.1167/iovs.07-0129 17962483

[B34] Averill MM Bornfeldt KE Lipids versus glucose in inflammation and the pathogenesis of macrovascular disease in diabetes Curr Diab Rep 2009 7 18 25 10.1007/s11892-009-0005-x 19192420 PMC3148110

[B35] He Z King GL Microvascular complications of *diabetes* Endocrinol Metab Clin North Am 2004 7 215 238 10.1016/j.ecl.2003.12.003 15053904

[B36] Ziyadeh FN Sharma K Overview: combating diabetic nephropathy J Am Soc Nephrol 2003 7 1355 1357 10.1097/01.ASN.0000065608.37756.58 12707405

[B37] Ishiyama-Shigemoto S Yamada K Yuan X Ichikawa F Nonaka K Association of polymorphisms in the beta2-adrenergic receptor gene with obesity, hypertriglyceridaemia, and diabetes mellitus Diabetologia 1999 7 1 98 101 10.1007/s001250051120 10027586

[B38] Nishio Y Kashiwagi A Kida Y Kodama M Abe N Saeki Y Shigeta Y Deficiency of cardiac beta-adrenergic receptor in streptozocin-induced diabetic rats Diabetes 1988 7 1181 1187 10.2337/diab.37.9.1181 2842211

[B39] Kamata K Miyata N Kasuya Y Involvement of endothelial cells in relaxation and contraction responses of the aorta to isoproterenol in naive and streptozotocin-induced diabetic rats J Pharmacol Exp Ther 1989 7 890 894 2543815

[B40] Kawamura T Egusa G Okubo M Imazu M Yamakido M Association of beta3-adrenergic receptor gene polymorphism with insulin resistance in Japanese-American men Metabolism 1999 7 1367 1370 10.1016/S0026-0495(99)90145-2 10582543

[B41] Walston J Lowe A Silver K Yang Y Bodkin NL Hansen BC Shuldiner AR The beta3-adrenergic receptor in the obesity and diabetes prone rhesus monkey is very similar to human and contains arginine at codon 64 Gene 1997 7 207 213 10.1016/S0378-1119(96)00796-2 9133593

[B42] Robertson K DNA methylation and human disease Nat Rev Genet 2005 7 597 610 16136652 10.1038/nrg1655

[B43] Jaenisch R Bird A Epigenetic regulation of gene expression: how the genome intergrates intrinsic and environmental signals Nat Genet 2003 7 245 254 10.1038/ng1089 12610534

[B44] Murrell A Rakyan VK Beck S From genome to epigenome Hum Mol Genet 2005 7 R3 R10 15809270 10.1093/hmg/ddi110

[B45] Hu S Wan J Su Y Song Q Zeng Y Nguyen HN Shin J Cox E Rho HS Woodard C Xia S Liu S Lyu H Ming GL Wade H Song H Qian J Zhu H DNA methylation presents distinct binding sites for human transcription factors Elife 2013 7 2 e00726 24015356 10.7554/eLife.00726 PMC3762332

[B46] Campanero MR Armstrong MI Flemington EK CpG methylation as a mechanism for the regulation of E2F activity Proc Natl Acad Sci USA 2000 7 6481 6486 10.1073/pnas.100340697 10823896 PMC18629

[B47] Wenger RH Kvietikova I Rolfs A Camenisch G Gassmann M Oxygen-regulated erythropoietin gene expression is dependent on a CpG methylation-free hypoxia-inducible factor-1 DNA-binding site Eur J Biochem 1998 7 771 777 10.1046/j.1432-1327.1998.2530771.x 9654078

[B48] Mulero-Navarro S Carvajal-Gonzalez JM Herranz M Ballestar E Fraga MF Ropero S Esteller M Fernandez-Salguero PM The dioxin receptor is silenced by promoter hypermethylation in human acute lymphoblastic leukemia through inhibition of Sp1 binding Carcinogenesis 2006 7 1099 1104 10.1093/carcin/bgi344 16410262

[B49] Emorine LJ Marullo S Delavier-Klutchko C Kaveri SV Durieu-Trautmann O Strosberg AD Structure of the gene for human beta 2-adrenergic receptor: expression and promoter characterization Proc Natl Acad Sci 1987 7 20 6995 6999 10.1073/pnas.84.20.6995 2823249 PMC299215

[B50] Mu S Shimosawa T Ogura S Wang H Uetake Y Kawakami-Mori F Marumo T Yatomi Y Geller DS Tanaka H Fujita T Epigenetic modulation of the renal β-adrenergic-WNK4 pathway in salt-sensitive hypertension Nat Med 2011 7 573 580 10.1038/nm.2337 21499270

[B51] Kong YH Zhang Y Li N Zhang L Gao YH Xue HJ Li Y Li WM Association between beta3-adrenergic receptor and oxidative stress in chronic heart failure rats Zhonghua Xin Xue Guan Bing Za Zhi 2010 7 5 435 439 20654104

[B52] Takahata Y Takarada T Iemata M Yamamoto T Nakamura Y Kodama A Yoneda Y Functional expression of beta2 adrenergic receptors responsible for protection against oxidative stress through promotion of glutathione synthesis after Nrf2 upregulation in undifferentiated mesenchymal C3H10T1/2 stem cells J Cell Physiol 2009 7 2 268 275 10.1002/jcp.21594 18814142

[B53] Steinle JJ Booz GW Meininger CJ Day JN Granger HJ Beta 3-adrenergic receptors regulate retinal endothelial cell migration and proliferation J Biol Chem 2003 7 20681 20686 10.1074/jbc.M300368200 12670949

[B54] Valko M Rhodes CJ Moncol J Izakovic M Mazur M Free radicals, metals and antioxidants in oxidative stress-induced cancer Chem Biol Interact 2006 7 1 1 40 10.1016/j.cbi.2005.12.009 16430879

[B55] Valko M Izakovic M Mazur M Rhodes CJ Telser J Role of oxygen radicals in DNA damage and cancer incidence Mol Cell Biochem 2004 7 1–2 37 56 15646026 10.1023/b:mcbi.0000049134.69131.89

[B56] Wachsman JT DNA methylation and the association between genetic and epigenetic changes: relation to carcinogenesis Mutat Res 1997 7 1 1 8 10.1016/S0027-5107(97)00003-1 9129674

